# The Role and Efficacy of Plasmapheresis in Acute Liver Failure Due to Hepatitis E Viral Hepatitis

**DOI:** 10.14309/crj.0000000000002160

**Published:** 2026-06-26

**Authors:** Sohail Hussain, Quratulain Khan, Fawad Hussain, Aimen Noor Ul Ain, Tauseef Mahmood, Faiza Nafees Khan

**Affiliations:** 1 Department of Gastroenterology, Ziauddin University, Karachi, Pakistan; 2 Department of Critical Care Medicine, Dr. Ziauddin University Hospital, Karachi, Pakistan; 3 Department of Nephrology, Ziauddin University Hospital, Karachi, Pakistan

**Keywords:** hepatitis E virus (HEV), acute liver failure (ALF), plasmapheresis, therapeutic plasma exchange (TPE), liver transplant alternative

## Abstract

Acute liver failure, due to hepatitis E virus (HEV) infection, represents a serious problem in developing countries, lacking access to liver transplantation. Therapeutic plasma exchange has become one potential bridge therapy, which has improved results by elimination of circulating toxins/inflammatory mediators, and ammonia. We describe the clinical course of a 31-year-old man who developed fulminant hepatic failure secondary to HEV infection. After conventional treatment, the patient underwent 5 sessions of plasmapheresis that was associated with marked biochemical and neurological recovery. This case provides support for plasmapheresis as a useful adjunct in the management of HEV-induced acute liver failure and its applicability in resource limited settings where liver transplantation is not available.

## INTRODUCTION

Acute liver failure (ALF) is defined as a rapid worsening of hepatic function in people without underlying preexisting liver disease with the presence of jaundice, coagulopathy (international normalized ratio > 1.5), and hepatic encephalopathy within 26 weeks of the onset of illness.^1–4^

The pathophysiology is dependent on the etiology of ALF. It occurs after primary injury to hepatocytes caused by hepatotropic viruses, hepatotoxic drugs, alcohol, or other hepatotoxicants which activate both the intrinsic and extrinsic cell-death pathways. Primary injury impairs Kupffer-cell tolerance and hence leads to activation and the release of proinflammatory mediators by these cells.

Despite major advances in critical care management, ALF is still linked to considerable morbidity and mortality, with mortality rates of between 60% and 80% when conventional treatment is used—especially in regions of the world where access to appropriate liver transplantation is limited.

Plasmapheresis or therapeutic plasma exchange (TPE) has gained attention as a supportive form of treatment for ALF. The European Association for the Study of the Liver and the American Association for the Study of Liver Diseases suggests consideration of TPE in ALF patients, particularly in whom liver transplantation is not an option.

Here, we report a case of hepatitis E virus (HEV)–induced ALF that was successfully treated with plasmapheresis and with results supported by recent studies showing its biochemical and beneficial survival effects.

## CASE REPORT

A 31-year-old man with no known comorbidities presented with fever, abdominal pain, jaundice, and altered level of consciousness. On physical examination, he had marked icterus without ascites or any stigmata of chronic liver disease.

The patient had been in good health until the sudden onset of symptoms. There was no history of alcohol use, and his body mass index was 23. He reported frequent consumption of street food several times per week.

His abdominal pain was severe and generalized, associated with recurrent vomiting and a high-grade fever (103°F). He initially sought care at a local clinic, where he received symptomatic treatment for pain relief. However, his symptoms persisted, and 1 day later, he developed irritability, restlessness, and behavioral changes, indicating worsening neurological status.

Owing to the progression of symptoms, he was subsequently brought to our hospital for further evaluation.

On arrival, the patient was drowsy with a Glasgow Coma Scale of 7/15, hypotensive (blood pressure 90/50 mm Hg), and in respiratory distress. He was electively intubated for airway protection, resuscitated with intravenous fluids, and subsequently transferred to the Medical Intensive Care Unit for further management. His Model for End-Stage Liver Disease score was 31, indicating severe hepatic dysfunction and high short-term mortality risk. Physical examination revealed marked icterus, with no evidence of ascites or other signs of chronic liver disease.

The initial laboratory findings are presented in Tables 1 and 2.

**Table 1.** The initial laboratory findings

Laboratory test	Result	Laboratory test	Result
Sodium (Na)	145 mEq/L	Ammonia	187 μmol/L
Potassium (K)	3.1 mEq/L	AST (SGOT)	3490 U/L
Chloride (Cl)	101 mEq/L	Total bilirubin	10.26 mg/dL
Bicarbonate (HCO_3_^−^)	23 mEq/L	Direct bilirubin	7.86 mg/dL
Hemoglobin (Hb)	15.7 g/dL	ALT (SGPT)	6684 U/L
PCV	48%	GGT	174 U/L
TLC	12.4 ×10^3^/µL	ALP	272 U/L
INR	3.66	Dengue NS1	Negative
Urea	18 mg/dL	MP-ICT	Negative
Creatinine	1.12 mg/dL	Procalcitonin (PCT)	0.0802 ng/mL
CRP	5.14 mg/L	Anti–HAV IgM (CLIA)^a^	Negative
		HBsAg (CLIA)^a^	Negative
		Anti-HCV (CLIA)^a^	Negative
		Anti–HEV IgM (CLIA)^a^	Positive

ALP, alkaline phosphatase; ALT, alanine aminotransferase; AST, aspartate transaminase; CLIA, chemiluminescence immunoassay; CRP, C-reactive protein; GGT, Gamma-glutamyl transferase; HAV, hepatitis A virus; HCV, hepatitis C virus; HEV, hepatitis E virus; ICT, indirect Coombs test; IgM, immunoglobulin M; INR, international normalized ratio; MP, Malaria parasite; PCV, packed cell volume; SGPT, serum glutamic-pyruvic transaminase; TLC, total leukocyte count.

^a^ Serological testing for viral hepatitis was performed using CLIA. Nucleic acid testing (HEV RNA) was not available in our setting.

**Table 2.** Trend of liver function tests and INR during plasma exchange

Parameter	Baseline	1st session	2nd session	3rd session	4th session	5th session
AST (SGOT) (U/L)	3,490	2,357	572	212	110	82
Total bilirubin (mg/dL)	10.26	8.20	5.91	5.68	3.89	3.78
INR	3.66	2.90	2.00	1.89	1.50	1.20
Direct bilirubin (mg/dL)	7.86	5.62	4.46	4.78	3.04	3.65
ALT (SGPT) (U/L)	6,684	5,027	1,527	694	407	336
GGT (U/L)	174	134	66	41	38	57
ALP (U/L)	272	174	127	94	80	76

ALP, alkaline phosphatase; ALT, alanine aminotransferase; AST, aspartate transaminase; GGT, Gamma-glutamyl transferase; INR, international normalized ratio; SGOT, serum glutamic-oxaloacetic transaminase; SGPT, serum glutamic-pyruvic transaminase.

Computed tomography of the brain did not show any evidence of ischemia, hemorrhage, or space-occupying lesions. Magnetic resonance imaging of the brain showed normal differentiation of the gray matter from white matter and no evidence of cerebral edema, hydrocephalus, or midline shift. Ultrasonographic examination of the abdomen revealed a liver of normal size and echotexture, without focal lesions or ascites; a mild right sided pleural effusion was however appreciated.

Based on the clinical presentation and the laboratory results, a diagnosis of ALF due to HEV infection was made. The patient was admitted to the Medical Intensive Care Unit and started on supportive management including intravenous N acetylcysteine, lactulose, rifaximin, vitamin K, and broad spectrum antibiotics. Intravenous fluids were given to achieve hemodynamic stabilization and airway protection was can be ensured by elective intubation due to hepatic encephalopathy. Despite initiation of optimal supportive therapy, no significant improvement was seen in neurological or biochemical parameters in 24 hours.

Consequently, a multidisciplinary consensus was reached, involving the transplant team, to initiate TPE as a rescue modality aimed at enhancing toxin clearance and improving hepatic function; if not, the patient would proceed to liver transplantation, given the limited time window for clinical intervention and the relatively delayed onset of action of antiviral therapy such as ribavirin.

After 2 sessions of plasmapheresis, the patient demonstrated a favorable response to treatment, with neurological improvement and a reduction in ventilatory requirement allowing extubation after 36 hours. In view of this positive clinical response, additional sessions were planned to get full recovery. The patient continued to improve clinically and was discharged on day 6, with normalized liver enzymes and complete resolution of encephalopathy.

## DISCUSSION

TPE or plasmapheresis has been recognized as a useful supportive therapy in ALF, especially in areas where liver transplantation is unavailable or delayed. The technique works by selectively eliminating circulating toxins, bilirubin, ammonia, and mediators of inflammation and simultaneously replacing vital proteins and coagulation factors in the plasma with donor plasma. Both the European Association for the Study of the Liver and the American Association for the Study of Liver Diseases state that TPE is a bridging therapy in patients awaiting transplantation or a supportive measure in nontransplant candidates.

The current case is very similar to those outcomes reported in these studies. After 5 sessions of TPE, the total bilirubin level in the patient fell from 10.26 to 3.78 mg/dL, alanine aminotransferase (SGPT) from 6,684 to 336 IU/L, and serum ammonia from 187 ug/dL to 32 ug/dL. This biochemical improvement was accompanied by a rapid neurological recovery with the Glasgow Coma Scale improving from 7/15 to 15/15 after the second session. The patient was successfully removed from the ventilator and was later released in good medical condition, which was the indication of successful hepatic regeneration without requiring a liver transplant.

The application of TPE for viral hepatitis-induced ALF has received more support in recent clinical studies. Maheshwari et al^5^ showed a statistically significant decrease in all the postprocedural values, such as total, direct, and indirect bilirubin; aspartate transaminase; alanine aminotransferase; prothrombin time; international normalized ratio; serum ferritin level; and Model for End-Stage Liver Disease score (*P* < 0.05).

Similarly, Gasca-Aldama et al^6^ demonstrated that TPE significantly increases 30-day survival in patients with ALF compared with the standard medical therapy only, thus favoring its use as an adjuvant therapy.

In line with these results, Bilgic et al^7^ also noticed significant decreases in total bilirubin and ammonia levels after 3 sessions of plasmapheresis in patients with viral hepatitis-related acute hepatic decompensation. Similarly, efficacy of plasmapheresis was reported from Pakistan with improvement in ammonia levels (from a baseline, 249 to 118 ug/dL at the third session).^8^ Our case was no different in showing a drop from baseline ammonia of 187 to 32 ug/dL at the fifth session.

Although the evidence to support the clinical efficacy of TPE in ALF is available, some limitations remain. Most existing data are obtained from observational studies and small case series, with little randomized controlled trials assessing its role in HEV-associated ALF. However, recent multicenter evidence with a study by Dong et al^9^ has contributed greatly to the growing evidence base. This was the first investigation conducted by the US ALF Study Group, and it showed that TPE improves short-term survival and biochemical recovery in patients with ALF who are not listed for transplantation. These findings improve patient selection, timing, and treatment protocols, offering higher quality evidence to incorporate TPE in the standard supportive care of ALF.

Overall, this case highlights the potential of early plasmapheresis in HEV induced ALF. Patient's rapid biochemical and neurological recovery stresses the importance of the therapy as a bridge not only to transplantation but also a bridge to recovery in centers that do not have transplant capability. Early diagnosis, early start of TPE, and multidisciplinary care and intensive care are crucial in improving survival outcome of this otherwise deadly disease. Further studies at large sample sizes are required to determine the precise effectiveness of plasmapheresis and to set standards against which healthcare providers can be measured.

## DISCLOSURES

Author contributions: S. Hussain: main consultant gastroenterologist and hepatologist involved in the patient's management; contributed to study conception, clinical interpretation, and critical revision of the manuscript for important intellectual content. Q. Khan: attending intensive care physician responsible for intensive care unit management; assisted in clinical data collection, interpretation, and manuscript editing. F. Hussain: assisted in patient management and clinical coordination; wrote the initial draft of the manuscript and contributed to the literature review. A. Noor Ul Ain: collected and organized patient data, conducted literature review, formatted references, and coordinated manuscript preparation and submission. T. Mahmood: assisted in preparing tables and figures, and contributed to data organization and presentation. F. Nafees Khan: reviewed and refined the final manuscript, provided expert input on methodology and clinical interpretation, and approved the final version for publication. S. Hussain is the article guarantor.

Financial disclosure: None to report.

Informed consent was obtained for this case report.
